# Discrepancies Between Bayesian Vancomycin Models Can Affect Clinical Decisions in the Critically Ill

**DOI:** 10.1155/2022/7011376

**Published:** 2022-11-17

**Authors:** Asad E. Patanwala, Danijela Spremo, Minji Jeon, Yann Thoma, Jan-Willem C. Alffenaar, Sophie Stocker

**Affiliations:** ^1^Faculty of Medicine and Health, School of Pharmacy, The University of Sydney, Camperdown, New South Wales, Australia; ^2^Department of Pharmacy, Royal Prince Alfred Hospital, Camperdown, New South Wales, Australia; ^3^REDS, School of Management and Engineering Vaud, HES-SO University of Applied Sciences and Arts, Delémont, Switzerland; ^4^Westmead Hospital, Westmead, New South Wales, Australia; ^5^Sydney Institute for Infectious Diseases, The University of Sydney, Sydney, New South Wales, Australia; ^6^Department of Clinical Pharmacology and Toxicology, St Vincent's Hospital, 390 Victoria Street, Darlinghurst, New South Wales 2010, Australia; ^7^St Vincent's Clinical Campus, University of NSW, Kensington, New South Wales, Australia

## Abstract

**Purpose:**

To assess the agreement in 24-hour area under the curve (AUC_24_) value estimates between commonly used vancomycin population pharmacokinetic models in the critically ill.

**Materials and Methods:**

Adults admitted to intensive care who received intravenous vancomycin and had a serum vancomycin concentration available were included. AUC_24_ values were determined using Tucuxi (revision cd7bd7a8) for dosing intervals with a vancomycin concentration using three models (Goti 2018, Colin 2019, and Thomson 2009) previously evaluated in the critically ill. AUC_24_ values were categorized as subtherapeutic (<400 mg·h/L), therapeutic (400–600 mg·h/L), or toxic (>600 mg·h/L), assuming a minimum inhibitory concentration of 1 mg/L. AUC_24_ value categorization was compared across the three models and reported as percent agreement.

**Results:**

Overall, 466 AUC_24_ values were estimated in 188 patients. Overall, 52%, 42%, and 47% of the AUC_24_ values were therapeutic for the Goti, Colin, and Thomson models, respectively. The agreement of AUC_24_ values between all three models was 48% (223/466), Goti-Colin 59% (193/466), Goti-Thomson 68% (318/466), and Colin-Thomson 67% (314/466).

**Conclusion:**

In critically ill patients, vancomycin AUC_24_ values obtained from different pharmacokinetic models are often discordant, potentially contributing to differences in dosing decisions. This highlights the importance of selecting the optimal model.

## 1. Introduction

International clinical practice guidelines and position statements suggest that the therapeutic drug monitoring of vancomycin should be guided by area under the curve (AUC) values [1–3]. This is because AUC-guided dosing is associated with less treatment failure and improved safety compared to trough-guided monitoring in patients with methicillin-resistant *Staphylococcus aureus* infection [4]. The ratio of the 24-hour AUC to the minimum inhibitory concentration (AUC_24_/MIC) is the pharmacokinetic (PK) parameter used to guide dosing decisions [1]. An AUC_24_/MIC target of 400–600 mg·h/L is considered to be optimal when MIC is determined by broth microdilution [1, 5]. In clinical practice actual MIC is often not available, and an MIC of 1 mg/L is assumed. Thus, an AUC_24_ <400 mg·h/L would prompt a dose increase to avoid treatment failure, and an AUC_24_ >600 mg·h/L would suggest a dose decrease is required to avoid acute kidney injury or other toxicity.

AUC_24_ can be estimated by first-order linear PK equations using two concentrations within a dose interval. This can be calculated manually or via readily available online calculators [6]. Alternatively, AUC_24_ can be estimated via population PK models using Bayesian software platforms [7]. The logistical advantage of the latter approach is that the AUC_24_ value estimate can be obtained using only one vancomycin concentration [6]. However, previous studies have shown a low precision of an AUC_24_ estimation using Bayesian software and a single concentration [8, 9]. In a cohort study of hospitalized patients (*n* = 978), clinical agreement was 76.8% between AUC_24_ values predicted by linear and Bayesian one-concentration [9]. The study was not specifically conducted among the critically ill. However, the investigators evaluated one Bayesian model [10] in the critically ill subset of the cohort.

Some of the Bayesian software platforms available offer multiple population PK models for the same medication, and end-users are required to select the most appropriate model. Thus, studies are needed to compare the clinical agreement between population PK models in the critically ill to inform this decision. It is possible that depending on the population PK model selected, it could lead to different dosing decisions. The objective of this study was to assess the agreement in AUC_24_ value estimates between commonly used vancomycin population PK models in the critically ill.

## 2. Methods

### 2.1. Ethics/Institutional Review Board

The study was approved by the Human Research Ethics committee of the Sydney Local Health District-Royal Prince Alfred Hospital zone (Approval number: 2019/ETH12033).

### 2.2. Study Design and Setting

This was a retrospective cohort study using data obtained from the intensive care unit (ICU) of a quaternary care hospital in Sydney, Australia. This is a substudy of a previously reported investigation evaluating the predictive performance of three vancomycin population PK models [8]. The hospital uses trough-based vancomycin monitoring to guide dosing decisions. Usually only one vancomycin concentration is obtained per dosing interval. Bayesian software is not used for clinical care, and AUC_24_ based monitoring has not been implemented. The ICU has a fully electronic medical record that includes all medications and pathology values used for the study (Philips IntelliSpace Critical Care and Anesthesia). Vancomycin concentrations are determined using an immunoassay, as has been previously described [8].

### 2.3. Participants and Data Acquisition

The study included adults (>18 years old) who were admitted to the ICU between 1 January 2019 and 31 May 2020, had received intravenous vancomycin via intermittent infusion, and had at least one vancomycin serum concentration available. Patients were excluded if they received continuous renal replacement therapy (CRRT) or a continuous infusion of vancomycin. Patients were also excluded if they were administered vancomycin doses on a non-ICU ward and subsequently transferred to the ICU, as dosing data were not available. All data were obtained from the electronic medical record. Vancomycin concentrations were categorized independently by two investigators as true trough concentrations or true troughs at steady state. A true trough concentration was defined as a concentration taken within 60 minutes before administration of an anticipated dose. A true trough at steady state was defined as a concentration taken within 60 minutes of the anticipated 4^th^ dose in treatment courses with <60-minute deviations from scheduled times for all prior doses.

### 2.4. Estimation of Vancomycin AUC24 Values

An AUC_24_ value was estimated for each dosing interval for which a vancomycin concentration was available using Tucuxi via a command line interface (revision cd7bd7a8 joint development by HEIG-VD, Yverdon-les-Bains, Switzerland and CHUV, Lausanne, Switzerland). All AUC24 value estimates were determined using a single vancomycin concentration. AUC_24_ values were estimated using three previously published vancomycin population PK models: (1) Goti [10], (2) Colin [11], and (3) Thomson [12]. Thus, each vancomycin concentration had three corresponding AUC_24_ value estimates.

### 2.5. Data Analysis

AUC_24_ values were categorized as subtherapeutic (<400 mg·h/L), therapeutic (400–600 mg·h/L), or toxic (>600 mg·h/L) for each model, assuming a minimum inhibitory concentration of 1 mg/L. These categorizations were defined and based on cut-off values from international guidelines [1, 3]. AUC_24_ value categorization was compared across the three models and reported descriptively as percent agreement. In other words, if an AUC_24_ value with one model was nontherapeutic and another model was therapeutic, then the two models would be discrepant and could result in different vancomycin dosing decisions. The concordance between AUC_24_ values from models was reported as scatter plots with the concordance correlation coefficient (*ρ*_*c*_) [13]. Other parameters calculated were Pearson's *r*, the average difference, the standard deviation of the difference, and Bland and Altman's 95% limits of agreement [14].

Two sensitivity analyses were conducted. First, the discrepancy between models was evaluated in the subset of concentrations that were considered as true troughs. Second, the discrepancy between models were evaluated in the subset of concentrations that were considered as true troughs at steady state. This was done as trough levels are commonly used in clinical practice, and it is possible that predictive performance is different in this subset [15]. All analyses were conducted in STATA 15 (College Station, Texas), and scatter plots were created using the R software (version 4.0.3, Vienna, Austria).

## 3. Results

### 3.1. Study Cohort

**Figure 1:**
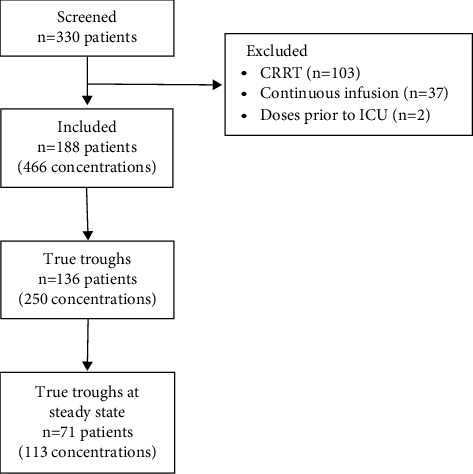
Flow diagram of sample selection. CRRT = continuous renal replacement therapy; ICU = intensive care unit.

A total of 330 patients received vancomycin during the study time frame and had a vancomycin concentration measured. Of these, 188 patients were included and had 466 vancomycin concentrations. The selection of the study cohort is shown in Figure 1. Of these, 250 concentrations were true troughs in 136 patients, and 113 concentrations were true troughs taken at steady state in 71 patients. The baseline characteristics of patients are in Table 1.

### 3.2. Main Results

The mean ± SD vancomycin AUC_24_ value was 469 ± 148 mg·h/L for Goti, 562 ± 172 mg·h/L for Colin, and 517 ± 164 mg·h/L for Thomson. Overall, 52%, 42%, and 47% of the AUC_24_ values were therapeutic for the Goti, Colin, and Thomson models, respectively.

Concordance between models is shown in Table 2. The agreement was 59% (*n* = 273/466) for Goti-Colin (*n* = 318/466), 68% for Goti-Thomson, and 67% (*n* = 314/466) for Colin-Thomson. Agreement between all three models was 48% (*n* = 223/466). The *ρ*_*c*_ was 0.75 for Goti-Colin, 0.77 for Goti-Thomson, and 0.81 Colin-Thomson. Other parameters are shown in Table 3.

**Figure 2:**
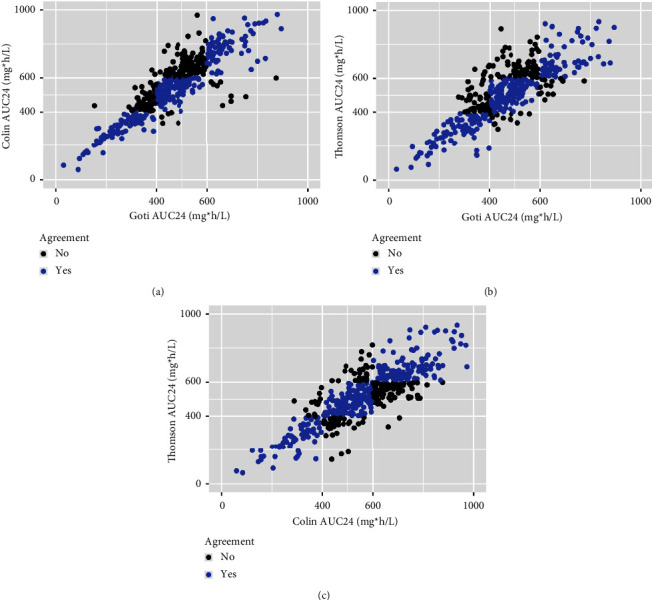
Scatter plot of AUC_24_ comparisons between models. Blue dots = agreement; black dots = no agreement; (a) Goti vs Colin models; (b) Goti vs Thomson models; and (c) Thomson vs Colin models.

The estimated AUC_24_ was more likely to be lower with Goti than with Colin or Thomson (Tables 2 and 3). In 14% (65/466) of the cases, Goti's estimate was subtherapeutic whereas Colin's was therapeutic or toxic, and in 24% (113/466) Goti's estimate was therapeutic, whereas Colin's was toxic. In 11% (51/466) Goti's estimate was subtherapeutic, whereas Thomson's was therapeutic or toxic, and in 14% (67/466) Goti's estimate was therapeutic, whereas Thomson's was toxic. Scatter plots of AUC_24_ comparisons between models are shown in Figure 2, and concordance parameters are shown in Table 3.

The two sensitivity analyses using the subset of concentrations that were true troughs or true troughs at steady state showed similar or lower agreement than the overall sample (Supplementary Appendix (available here)). Overall, agreement for true trough subset was 48% between all models (*n* = 121/250), and true trough at steady state subset was 42% between all models (*n* = 47/113). Parameters for concordance between models and scatterplots for the sensitivity analysis subsets are in the Supplementary Appendix (available here). There were 37 concentrations that were therapeutic (15–20 mg/L) and considered true troughs at steady state. Of these, 28 (75%) in the Goti model, 8 (22%) in the Colin model, and 20 (54%) in the Thomson model had estimates of a therapeutic AUC_24_ value.

## 4. Discussion

The key finding of this investigation was that agreement between vancomycin PK models occurred approximately two-thirds of the time. In other words, clinicians may come to different dosing decisions based on standard cut-off values for AUC_24_ in 1 of 3 ICU patients, depending on the PK model used. The Goti model had lower AUC_24_ estimates than Colin and Thomson. Thus, using Goti-based AUC_24_ estimates, clinicians would be prompted to use higher doses than the other two models.

The question regarding the most appropriate PK model for the critically ill has not been settled. Some commonly used Bayesian software platforms have recommended the Goti model for ICU patients [9]. Although the three models used in our investigation were not derived using critically ill patients exclusively, they have been subsequently evaluated in ICU cohorts [8, 16, 17]. In a retrospective study, data from 82 ICU patients was used to evaluate 12 vancomycin PK models [16]. The investigators considered a model to be clinically acceptable if the relative bias was ±20% and the 95% CI included zero. The Goti model was the only one considered to be clinically acceptable based on both *a priori* and *a posteriori* approaches. However, the definition for acceptability did not include parameters for precision. In a larger study (*n* = 188 patients) of the same cohort as our current investigation [8], it appeared that the Goti model may be slightly more suitable based on precision, but the extent of the differences between PK models were too small to be clinically meaningful. Another smaller investigation with data from 50 patients and using simulation techniques considered the model by Thomson to have the best predictive performance [17]. However, precision appeared to be low based on the relative root mean squared error reported by the authors. In addition, the model by Goti was not evaluated in the aforementioned study. Vancomycin PK models need to be improved for suitable precision of AUC_24_ estimated from one concentration. It is possible that sparse sampling has been a contributor to models with poor precision.

The AUC_24_ can also be estimated by taking two concentrations within a dose interval (e.g., peak and trough levels). The estimation is based on first-order PK equations. A detailed approach to these calculations has been described [18]. Although this can be done manually, readily available software programs can be used to conduct these calculations with relatively little training [6]. AUC_24_ estimation via first order PK calculations using two levels is the reference standard to which Bayesian estimates have been compared [9]. Such a comparison has been described in a retrospective cohort study [9] (*n* = 978 patients) where clinical agreement was assessed between first-order PK equation-calculated AUC_24_ vs Bayesian two- (i.e., peak and trough) and one-concentration AUC_24_ (i.e., trough only). Clinical agreement was defined similarly to the cut-off values in our study. The PK model used for Bayesian estimation depended on the patient (noncritically ill, critically ill, and obese). The Goti model was used in the critically ill subset and 69% of the patients were critically ill. Clinical agreement was higher with two-concentration estimates (87.4%) than one-concentration (76.8%) estimates. In clinical practice, the advantage of the Bayesian AUC_24_ is that it can be estimated using just one concentration. Thus, the results pertaining to the one-concentration estimate are more relevant. If two concentrations are taken, then clinicians could use the reference standard first-order PK equations, and Bayesian estimates are not needed. The clinical agreement using one-concentration Bayesian AUC_24_ was still higher than the agreement we found between PK models in our study. This may be because there was less variability in the noncritically ill patients in the aforementioned study that made up approximately one-third of the patient subset.

Sensitivity analyses were conducted to evaluate subsets of patients with true troughs and true troughs at steady state. We considered that some PK models are developed using routine clinical data rather than richly sampled data with multiple concentrations taken during a dosing interval. This is true for the Goti model, where most patients had only one sample taken [10]. These are usually trough concentrations (or similar). Thus, prediction using such a PK model may be different for troughs. In addition, trough concentrations are more likely to reflect what is done in clinical practice. However, we did not show that agreement between PK models changed in the subsets with true troughs.

Given the uncertainty of the evidence, the availability of different PK models within Bayesian software platforms, and the possible lack of agreement between PK models, we suggest that if clinicians use a one-concentration Bayesian estimation using currently available PK models, it may be useful to verify the estimation using two different PK models available within the software program (e.g., Goti and Thomson). This can be done relatively quickly and does not require the re-entry of patient data into the software platforms we are aware of. A mid-interval concentration may also improve estimates [15]. The discrepancies in the estimate and direction of the discrepancy could help guide dosing decisions, which must be made in the context of the clinical situation. For example, a subtherapeutic AUC_24_ with the Goti model but a therapeutic AUC_24_ with the Thomson model would still support a dose increase if an infection was severe or the patient was deteriorating. Based on the uncertainty and lack of precision, our institution has not implemented the use of Bayesian software platforms. Instead, there are ongoing efforts to transition to AUC_24_ estimation using two concentrations calculated from first-order PK equations in the ICU.

Our study has some limitations. First, we have only compared AUC_24_ estimates between PK models. From the data available we were unable to determine the “true” AUC value as a reference. As such, we are unable to comment on the accuracy of AUC value estimates derived from each of the population PK models. Second, we did not assess the implication of drug exposure on clinical outcomes. However, that was not the intent of this study. An evaluation of clinical outcomes would require a comprehensive clinical dataset to adjust for any potential confounders as well as an appropriate assessment of the drug concentration and pathogen susceptibility [19]. Third, ICU patients are a heterogenous population and it is possible that clinical agreement would be improved in some subsets or phenotypes of patients. However, we do not have the data to meaningfully delineate this.

## 5. Conclusion

In critically ill patients, vancomycin AUC_24_ values estimated from different PK models are often discordant, potentially contributing to differences in dosing decisions. This highlights the importance of selecting the optimal model. Given the lack of agreement between PK models currently used in Bayesian software, it may be useful to use more than one model to guide decisions that are supported by clinical context, especially when estimates are close to decision cut-off values for AUC_24_.

## Figures and Tables

**Table 1:** Patient demographics.

Demographics	Full sample *n*_p_ = 188 *n*_c_ = 466	True trough *n*_p_ = 136 *n*_c_ = 250	True trough at steady state *n*_p_ = 71 *n*_c_ = 113
Age (years), mean (SD)	58 (17)	59 (17)	59 (17)
Sex (male), *n* (%)	119 (63)	89 (65)	48 (68)
APACHE III score, mean (SD)	62 (22)	62 (21)	64 (22)
Mechanical ventilation, *n* (%)	74 (39)	60 (44)	34 (45)
Vasopressors, *n* (%)	66 (35)	48 (35)	33 (46)

*n*_p_ = number of patients; *n*_c_ = number of concentrations; SD = standard deviation; APACHE = acute physiology and chronic health evaluation.

**Table 2:** Agreement between models at decision cut-offs.

	AUC_24_ mg·h/L	<400 *n* (%)	400–600 *n* (%)	>600 *n* (%)	Total
Agreement = 59% (273/466)					
	Goti				
Colin	<400	75 (16)	5 (1)	0 (0)	80
	400–600	63 (14)	122 (26)	10 (2)	195
	>600	2 (<1)	113 (24)	76 (16)	191
	Total	140	240	86	466

*Agreement* *=* *68% (318/466)*					
	Goti				
Thomson	<400	89 (19)	14 (3)	0 (0)	103
	400–600	46 (10)	159 (34)	16 (3)	221
	>600	5 (1)	67 (14)	70 (15)	142
	Total	140	240	86	466

*Agreement* *=* *67% (314/466)*					
	Colin				
Thomson	<400	67 (14)	34 (7)	2 (<1)	103
	400–600	13 (3)	133 (29)	75 (16)	221
	>600	0 (0)	28 (6)	114 (24)	191
	Total	80	195	191	466

**Table 3:** Concordance between models.

	Goti-Colin	Goti-Thomson	Colin-Thomson
*ρ*_c_	0.75	0.77	0.81
Pearson's *r*	0.88	0.81	0.84
Difference			
Average (mg·h/L)	−93	−48	45
Standard deviation (mg·h/L)	82	98	97
95% LOA (mg·h/L)	−253–68	−239–144	−145–235

*ρ*_c_ = concordance correlation coefficient; LOA = Bland and Altman's limits of agreement.

## Data Availability

Data are available upon reasonable request to the corresponding author.

## Additional Points

What is already known about this subject: Monitoring of vancomycin should be guided by 24-hour area under the curve (AUC_24_). AUC_24_ can be estimated via population pharmacokinetic (PK) models using Bayesian software platforms. Different vancomycin population PK models are available for AUC_24_ estimation. What this study adds: Vancomycin AUC_24_ values obtained from different PK models were often discordant at decision thresholds. Clinical dosing decisions of vancomycin may be dependent on the PK model used.

## Supplementary Materials

Supplementary Materials Table S1: Cross-tabulation of agreement between models at decision cut-offs in the subset with true troughs. Table S2: Cross-tabulation of agreement between models at decision cut-offs in the subset with true troughs at steady state. Table S3: Concordance parameters between models in the sensitivity analysis subsets. Figure S1: Scatterplot of AUC24 between models in subset of concentrations that were true troughs. Figure S2: Scatterplot of AUC24 between models in subset of concentrations that were true troughs at steady state.

## References

[1] Matsumoto K., Oda K., Shoji K. (2022). Clinical practice guidelines for therapeutic drug monitoring of vancomycin in the framework of model-informed precision dosing: a consensus review by the Japanese society of chemotherapy and the Japanese society of therapeutic drug monitoring. *Pharmaceutics*.

[2] Reuter S. E., Stocker S. L., Alffenaar J. W. C. (2022). Optimal practice for vancomycin therapeutic drug monitoring: position statement from the anti-infectives committee of the international association of therapeutic drug monitoring and clinical toxicology. *Therapeutic Drug Monitoring*.

[3] Rybak M. J., Le J., Lodise T. P. (2020). Therapeutic monitoring of vancomycin for serious methicillin-resistant *Staphylococcus aureus* infections: a revised consensus guideline and review by the American society of health-system pharmacists, the infectious diseases society of America, the pediatric infectious diseases society, and the society of infectious diseases pharmacists. *American Journal of Health-System Pharmacy*.

[4] Tsutsuura M., Moriyama H., Kojima N. (2021). The monitoring of vancomycin: a systematic review and meta-analyses of area under the concentration-time curve-guided dosing and trough-guided dosing. *BMC Infectious Diseases*.

[5] Rybak M. J. (2006). The pharmacokinetic and pharmacodynamic properties of vancomycin. *Clinical Infectious Diseases*.

[6] Sanford G. (2022). Vancomycin Calculator. https://www.sanfordguide.com/vancomycin-dosing/.

[7] Drennan P. G., Doogue M., van Hal S. J., Chin P. (2018). Bayesian therapeutic drug monitoring software: past, present and future. *International Journal of Pharmacokinetics*.

[8] Narayan S. W., Thoma Y., Drennan P. G. (2021). Predictive performance of bayesian vancomycin monitoring in the critically ill. *Critical Care Medicine*.

[9] Olney K. B., Wallace K. L., Mynatt R. P. (2022). Comparison of bayesian-derived and first-order analytic equations for calculation of vancomycin area under the curve. *Pharmacotherapy: The Journal of Human Pharmacology and Drug Therapy*.

[10] Goti V., Chaturvedula A., Fossler M. J., Mok S., Jacob J. T. (2018). Hospitalized patients with and without hemodialysis have markedly different vancomycin pharmacokinetics: a population pharmacokinetic model-based analysis. *Therapeutic Drug Monitoring*.

[11] Colin P. J., Allegaert K., Thomson A. H. (2019). Vancomycin pharmacokinetics throughout life: results from a pooled population analysis and evaluation of current dosing recommendations. *Clinical Pharmacokinetics*.

[12] Thomson A. H., Staatz C. E., Tobin C. M., Gall M., Lovering A. M. (2009). Development and evaluation of vancomycin dosage guidelines designed to achieve new target concentrations. *Journal of Antimicrobial Chemotherapy*.

[13] Lin L. I. K. (1989). A concordance correlation coefficient to evaluate reproducibility. *Biometrics*.

[14] Martin Bland J., Altman D. G. (1986). Statistical methods for assessing agreement between two methods of clinical measurement. *The Lancet*.

[15] Shingde R. V., Reuter S. E., Graham G. G. (2020). Assessing the accuracy of two bayesian forecasting programs in estimating vancomycin drug exposure. *Journal of Antimicrobial Chemotherapy*.

[16] Cunio C. B., Uster D. W., Carland J. E. (13 2021). Towards precision dosing of vancomycin in critically ill patients: an evaluation of the predictive performance of pharmacometric models in ICU patients. *Clinical Microbiology and Infections*.

[17] Heine R., Keizer R. J., Steeg K. (2020). Prospective validation of a model-informed precision dosing tool for vancomycin in intensive care patients. *British Journal of Clinical Pharmacology*.

[18] DeRyke C. A., Alexander D. P. (2009). Optimizing vancomycin dosing through pharmacodynamic assessment targeting area under the concentration-time curve/minimum inhibitory concentration. *Hospital Pharmacy*.

[19] Martson A. G., Sturkenboom M. G. G., Stojanova J. (2020). How to design a study to evaluate therapeutic drug monitoring in infectious diseases?. *Clinical Microbiology and Infections*.

