# Supplementary material for: Discrepancies Between Bayesian Vancomycin Models Can Affect Clinical Decisions in the Critically Ill
Source: Crit Care Res Pract. 2022 Nov 17;2022:7011376. doi: 10.1155/2022/7011376 (PMC9767744; doi:10.1155/2022/7011376)
Supplement: Supplementary Materials — Table S1: Cross-tabulation of agreement between models at decision cut-offs in the subset with true troughs. Table S2: Cross-tabulation of agreement between models at decision cut-offs in the subset with true troughs at steady state. Table S3: Concordance parameters between models in the sensitivity analysis subsets. Figure S1: Scatterplot of AUC24 between models in subset of concentrations that were true troughs. Figure S2: Scatterplot of AUC24 between models in subset of concentrations that were true troughs at steady state. [file 7011376.f1.docx]

**Supplementary Appendix**

Table S1. Agreement between models at decision cut-offs in subset with true troughs

| Agreement = 60% (150/250) | | | | |
| --- | --- | --- | --- | --- |
|  | Goti | | | |
| Colin | AUC_24_  mg⋅h/L | <400 | 400-600 | >600 |
|  | <400 | 37 | 2 | 0 |
|  | 400-600 | 31 | 70 | 5 |
|  | >600 | 0 | 62 | 43 |
| Agreement = 67% (167/250) | | | | |
|  | Goti | | | |
| Thomson | AUC_24_  mg⋅h/L | <400 | 400-600 | >600 |
|  | <400 | 39 | 7 | 0 |
|  | 400-600 | 28 | 88 | 8 |
|  | >600 | 1 | 39 | 40 |
| Agreement = 70% (174/250) | | | | |
|  | Colin | | | |
| Thomson | AUC_24_  mg*h/L | <400 | 400-600 | >600 |
|  | <400 | 31 | 15 | 0 |
|  | 400-600 | 8 | 77 | 39 |
|  | >600 | 0 | 14 | 66 |

Table S2. Agreement between models at decision cut-offs in subset with true troughs at steady state

| Agreement = 54% (61/113) | | | | |
| --- | --- | --- | --- | --- |
|  | Goti | | | |
| Colin | AUC_24_  mg⋅h/L | <400 | 400-600 | >600 |
|  | <400 | 8 | 1 | 0 |
|  | 400-600 | 9 | 30 | 2 |
|  | >600 | 0 | 40 | 23 |
| Agreement = 65% (74/113) | | | | |
|  | Goti | | | |
| Thomson | AUC_24_  mg⋅h/L | <400 | 400-600 | >600 |
|  | <400 | 9 | 3 | 0 |
|  | 400-600 | 8 | 45 | 5 |
|  | >600 | 0 | 23 | 20 |
| Agreement = 64% (72/113) | | | | |
|  | Colin | | | |
| Thomson | AUC_24_  mg*h/L | <400 | 400-600 | >600 |
|  | <400 | 6 | 6 | 0 |
|  | 400-600 | 3 | 29 | 26 |
|  | >600 | 0 | 6 | 37 |

Table S3. Concordance between models in sensitivity analysis subsets

| *Subset of concentrations that were true troughs* | | | |
| --- | --- | --- | --- |
|  | Goti-Colin | Goti-Thomson | Colin-Thomson |
| ρ_c_ | 0.75 | 0.77 | 0.82 |
| Pearson’s r | 0.90 | 0.82 | 0.84 |
| Difference |  |  |  |
| Average (mg·h/L) | -97 | -54 | 43 |
| Standard deviation (mg·h/L) | 78 | 96 | 97 |
| 95% LOA (mg·h/L) | -250 – 57 | -243 – 134 | -148 – 233 |
| *Subset of concentrations that were true troughs at steady state* | | | |
| ρ_c_ | 0.65 | 0.66 | 0.70 |
| Pearson’s r | 0.86 | 0.73 | 0.74 |
| Difference |  |  |  |
| Average (mg·h/L) | -109 | -59 | 51 |
| Standard deviation (mg·h/L) | 80 | 109 | 113 |
| 95% LOA (mg·h/L) | -268 – 49 | -272 – 154 | -170 – 271 |

ρ_c_ = concordance correlation coefficient; LOA = Bland and Altman’s Limits of Agreement

Figure S1. Scatterplot in Subset of concentrations that were true troughs

Figure S2. Scatterplot in Subset of concentrations that were true troughs at steady state
